# Conjunctival ultraviolet autofluorescence as a biomarker of outdoor time in myopic children

**DOI:** 10.3389/fmed.2024.1492180

**Published:** 2024-12-09

**Authors:** Miriam de la Puente, Valentina Bilbao-Malavé, Jorge González-Zamora, Aura Ortega Claici, Jaione Bezunartea, Leire Gomez-Arteta, Elena Alonso, María Hernández, Patricia Fernández-Robredo, Manuel Sáenz de Viteri, Nerea Martín Calvo, Alfredo García-Layana, Jesús Barrio-Barrio, Sergio Recalde

**Affiliations:** ^1^Department of Ophthalmology, Clínica Universidad de Navarra, Pamplona, Spain; ^2^Retinal Pathologies and New Therapies Group, Experimental Ophthalmology Laboratory, Department of Ophthalmology, Universidad de Navarra, Pamplona, Spain; ^3^Department of Ophthalmology, Bellvitge University Hospital, Barcelona, Spain; ^4^Faculty of Medicine, Universidad de Navarra, Pamplona, Spain; ^5^Navarra Institute for Health Research, IdiSNA, Pamplona, Spain; ^6^Red Temática de Investigación Cooperativa en Salud: ‘Prevention, Early Detection, and Treatment of the Prevalent Degenerative and Chronic Ocular Pathology’ from (RD16/0008/0011), Ministerio de Ciencia, Innovación y Universidades, Instituto de Salud Carlos III, Madrid, Spain; ^7^School of Medicine, Department of Preventive Medicine and Public Health, University de Navarra, Pamplona, Spain; ^8^CIBERobn Physiopathology of Obesity and Nutrition, Carlos III Institute of Health, Madrid, Spain

**Keywords:** myopia, light exposure, outdoor activities for children, CUVAF, biomarker

## Abstract

**Introduction:**

The prevalence of myopia has increased significantly in recent years including an earlier onset of myopia development on the pediatric population. The main objective of the study is to compare CUVAF (Conjunctival Ultraviolet Autofluorescence) in children with and without myopia to validate its usefulness as an outdoor protective biomarker.

**Methods:**

A case–control observational study was conducted in a child cohort from subjects that attended to the Ophthalmology Department of Clínica Universidad de Navarra for an ophthalmological examination. The general exclusion criteria were (among others): amblyopia, congenital myopia, general ophthalmic disease, and any conjunctival alteration that might difficult the measurement of the CUVAF area. All participants underwent an automatic objective refraction under cycloplegic effect, biometry to measure axial length (AL) and central corneal radius (CCR), and completed a questionnaire about their lifestyle habits. A total of 4 images of the bulbar conjunctiva were taken with blue light in order to quantify the CUVAF area.

**Results:**

A total of 263 subjects (6 to 17 years old) were analyzed with no significant differences in demographic data between case group and control group. There were 50 non-myopic subjects (19%) and 213 myopic subjects (81%). In relation to the outdoor activities (OA), myopic subjects spent significantly fewer hours per week outdoors than the control-group (*p* = 0.03). About the CUVAF area, the differences between groups were statistically significant, showing that the myopic group has a significantly smaller CUVAF area than the control-group (0.33 ± 0.72 mm^2^ vs. 0.78 ± 1.22 mm^2^; *p* = 0.0023), likewise, the frequency of CUVAF area absence between both groups showed an odds ratio (OR) of 2.52 (CI95% 1.33–4.74). A Pearson correlation test was done, obtaining a strong significant inverse correlation between myopia degree-CUVAF area (*r* = 0.1877; IC95% 0.068–0.302), and also ratio (AL/CCR)-CUVAF area (*p* = 0.002 and *p* = 0.04) respectively.

**Conclusion:**

CUVAF is a useful biomarker for OA and it has an inverse relationship with myopia degree also in pediatric age, especially after the age of 12, so it could be useful to differentiate the risk of developing myopia. Having a CUVAF area greater than that corresponding to age, protect to myopia 2.5 times, being almost 5 times the protection in case of high myopia.

## Introduction

Nowadays, myopia is the most prevalent refractive error and its increase is considered an important cause of concern in the population (1–4). In fact, over the last decades, has undergone a very significant increase in prevalence worldwide, especially in Asia. (5). Globally, it is estimated that almost 50% of the population will be myopic by 2050 (6) and according to World Health Organization (WHO) estimations, 2.8% of the general population had high myopia (HM) in 2010, and this percentage could reach 10% by 2050 (6–11).

Regarding children, the highest prevalence of myopia using cycloplegic refraction examinations was reported in East Asia and Singapore (60%), while in Europe the prevalence rates were lower (40%) (2, 12). Being the most sensitive age of myopia growth between 7 and 10 years old when it increased 5.1 times (from 4.5 to 23.0%), 15–18 years increased 2.1 times (from 21.5 to 45.0%), stabilizing from the age of 24, except in high myopic patients (13).

The relevance of this data is that early-onset myopia generally progresses through adulthood, leading to an increase in HM prevalence, which is a well known risk factor for the development of other ocular pathologies, such as glaucoma, cataract, retinal detachment or myopic maculopathy (14–16). Because of that reason, the prevention of myopia and HM has been considered by the WHO as one of its five priorities in the global prevention of blindness (17).

The complexity of myopia results from the fact that it is not caused by a single etiological factor. Some risk factors are well known, particularly genetic and environmental factors, although it has not been possible to determine the degree of influence of each factor in each patient (16, 18–20). Among the modifiable factors, time spent doing outdoor activities (OA), as a protective factor, and near vision activities (NVA), as a risk factor, are clearly the most important (16, 20–29).

The most frequently employed methodology in epidemiological studies for determining an individual’s time spent doing OA is the use of questionnaires (10, 11, 30, 31). However, this data may not be accurate due to recall bias, inaccuracy of questions or errors of interpretation (10, 21, 32), which may result in overestimation or underestimation of the actual or past time spent outdoors. Given the shifting population habits and the necessity for conducting objective research on myopia and its associated risk and protective factors, it is clear that an objective biomarker is needed. Recently, CUVAF (Conjunctival Ultraviolet Autofluorescence) area measurement, has been studied for this purpose (33). It was first described as a localized-area of autofluorescence under ultraviolet (UV) light in the bulbar conjunctiva (34–36), secondary to sunlight exposure (37–39). To detect CUVAF-area, a special custom-built device was designed initially, and subsequently, Lingham et al. validated the autofluorescence mode of the Heidelberg Spectralis HRA + OCT (optical coherence tomography), a device commonly used in ophthalmology clinics for other purposes (34, 40). CUVAF is based on the premise that conjunctival components may emit visible fluorescence as their structure is altered by UV exposure. Changes in intracellular protein content in endogenous cellular components, such as lysosomes, mitochondria, cytokines, growth factors and matrix metalloproteinases (MMPs), are also implicated in the pathogenesis of pterygium (39, 41).

Previous studies conducted by our research group with university students have demonstrated a negative correlation between the spherical equivalent (SE) of the subjects and the amount of time spent doing OA in myopic subjects (10, 42). This relationship has also been observed in children, with the CUVAF area increasing with greater sun exposure (taking into account the amount of exposed skin), and also increasing with age (43). Additionally, there is an inverse correlation between the individual’s refractive error (RE) and the CUVAF-area (10, 44). These results have been corroborated in a meta-analysis, which aimed to provide a comprehensive summary of the relevant evidence pertaining to the association between the CUVAF-area and myopia across different geographic regions and age groups (33).

To date, studies in this field have been conducted primarily in adults and young adults, however, the utility of CUVAF as an objective biomarker of myopia and its risk/protective factors in the pediatric population remains poorly understood. The objective of this study is to compare the CUVAF in children with and without myopia in order to validate its usefulness as a protective biomarker of childhood myopia.

## Materials and methods

### Study design and ethics approval

A case–control observational study was conducted in a child population forming a homogeneous sample in which there were no differences in terms of age or gender. The project was approved by the Ethics Committee (study code 2021.083) of the Clínica Universidad de Navarra. The subjects belonging to the control group were obtained from routine clinical check-ups attended by the patients in which no pathology was found.

All of the subjects that were included in the study were fully aware of the purpose and procedures of the study and informed consent was obtained from the legal guardians of all participants.

### Inclusion and exclusion criteria

The inclusion criteria was subjects that attended to the Ophthalmology Department of Clínica Universidad de Navarra aged between 6 and 17 years old. The exclusion criteria were astigmatism or anisometropia ≥2.0 diopters (D), amblyopia, congenital myopia or myopia associated to another pathology, other ophthalmic disease that may affect visual acuity or interfere with the performance of tests, as well as any conjunctiva alteration that might difficult the measurement of the CUVAF-area.

The participants were classified into control or myopic according to their SE. The myopic patients were subclassified according to their degree of myopia in “Low myopia” (M1; −0.75 to −2.75 D), “Moderate myopia” (M2; −3 to −5.75 D) and “High myopia” [HM; ≤ − 6 D or ≥ 26 mm of axial length (AL)]. After that, a division by subgroups was made, separating the patients into those under and over 12 years old. This limit was selected because of the change of activities at this age as well as some hormonal changes that occur due to the onset of adolescence (45, 46), which may also play an important role in the development of myopia.

### Data collection

All participants underwent an automatic objective refraction under cycloplegic effect with three drops of Cyclopentolate 1% every 10 min, and the measurements were taken 45 min after the last drop (Autorefractor Keratometer TRK-2P. Topcon Corporation, Tokyo, Japan), biometry to measure AL and central corneal radius (CCR), which are then used to obtain an index of the ratio between AL and CCR (IOLMaster; Carl Zeiss Meditec, Jena, Germany). This value was only measured in patients in the second inclusion phase, so only 163 measurements are available. Participants (under legal guardians supervision) were also asked to complete a questionnaire about their family history of myopia (categorical variables; CV), spectacles or contact lenses use (CV), time spent doing NVA and OA during a regular week (continues variables), and sun exposure habits (CV). Furthermore, a total of 4 images (nasal and temporal image of each eye) of the bulbar conjunctiva were taken from every patient with the blue autofluorescence (BAF) module on the Heidelberg Spectralis HRA + OCT (Heidelberg Engineering, Heidelberg, Baden-Württemberg, Germany), using the image acquisition protocol recently validated by Lingham et al. (40).

Quantification of the CUVAF area was performed using a plugin developed by the imaging platform of the Center for Applied Medical Research (CIMA) of the University of Navarra using Fiji/ImageJ 1.6v (NIH, Bethesda, MD, USA), an open source Java-based image processing software. Three different investigators performed CUVAF-area measurement and the intra and inter-observer reliability between them was evaluated. The mean area of the 4 images of each subject was used for the posterior analysis.

### Statistical analyses

All the information was stored in accordance with data protection laws and grouped into variables in a database for subsequent analysis. After testing the normal distribution of the sample, the general characteristics of the participants were compared using Student’s t-test and one-criterion ANOVA for continuous variables and Fisher’s F test for categorical variables. Pearson correlation tests were performed between the variables CUVAF-area and SE, AL, CCR and AL/CCR ratio.

For all statistical analyses, the *α*-error had been previously established as a *p* < 0.05 (two-tailed). Statistical analyses were performed using SPSS 20.1 Software (SPSS Inc., Chicago, IL, USA) and GraphPad Prism Software version 5.0 (GraphPad Software Inc., San Diego, CA, USA).

## Results

### Demographic characteristics

In total, 283 patients were analyzed and, after the evaluation of inclusion and exclusion criteria, 263 patients were included in the study. Table 1 summarizes the participant’s data and the characteristics of each group (controls, M1, M2 and HM).

**Table 1:** Data obtained on study participants about their demographic, ophthalmologic, CUVAF and environmental factors.

	Total	Controls SE > -0.75 D	M1-0.75 to −2.75 D	M2-3 to −5.75 D	HM ≤ -6 D or ≥ 26 mm of AL	*p* value
Number (%)	263 (100%)	50 (19%)	134 (51%)	51 (19%)	28 (11%)	–
Age (mean ± SD)	12.60 ± 2.82	12.54 ± 2.89	12.20 ± 3.05	13.18 ± 2.23	13.55 ± 2.15	0.26
Female (%)	105 (40%)	15 (30%)	49 (36.3)	30 (59)	11 (39)	0.23
Myopia onset age (mean ± SD)	8.23 ± 3,03	–	9.39 ± 2.67	6.86 ± 2.47****	6.39 ± 2.62****	<0.0001
SE (D ± SD)	−2.34 ± 2.11	0.17 ± 1.33	−1.79 ± 0.76****	−4.22 ± 0.71****	−6.02 ± 1.54****	<0.0001
AL (mm ± SD)	24.29 ± 0.99	23.58 ± 0.79	24.22 ± 0.69****	24.82 ± 0.69****	26.22 ± 0.49****	<0.0001
CUVAF (mm^2^ ± SD)	0.42 ± 0.85	0.78 ± 1.22	0.41 ± 0.78*	0.22 ± 0.68**	0.16 ± 0.40**	0.0023
Near act. Hours/week (± SD)	29.82 ± 10.97	30.22 ± 14.90	28.44 ± 11.62	32.91 ± 7.35	28.69 ± 8.81	0.20
Outdoor act. Hours/week (± SD)	10.81 ± 4.18	14.28 ± 5.69	10.92 ± 4.39*	10.33 ± 3.43*	10.47 ± 2.79*	0.025
Ratio (AL/CCR) (mean ± SD)	3.06 ± 0.19	2.88 ± 0.26	2.99 ± 0.17	3.21 ± 0.08**	3.27 ± 0.14**	0.0006
CUVAF > 0 (%)	81 (31%)	24 (48%)	44 (33%)	8 (16%)***	5 (18%)**	0.006
Both parents with myopia (%)	–	20%	32%*	25%	41%**	0.0075
No parents with myopia (%)	–	29%	18%*	11%**	11%**	0.0017

First row includes cohort classification based on SE *p < 0.05; **p < 0.01; ****p < 0.0001. ANOVA or F Fisher value. Comparisons are between Control or M1 group vs. the other groups. Standard Deviation (SD), Spherical Equivalent (SE), Diopters (D), Axial Length (AL), Conjunctival Ultraviolet Autofluorescence (CUVAF), Central corneal radius (CCR). Data for subjects who only have one parent with myopia are not included in the last rows of the table, so they do not add up to 100% of the participants.

**Figure 1:**
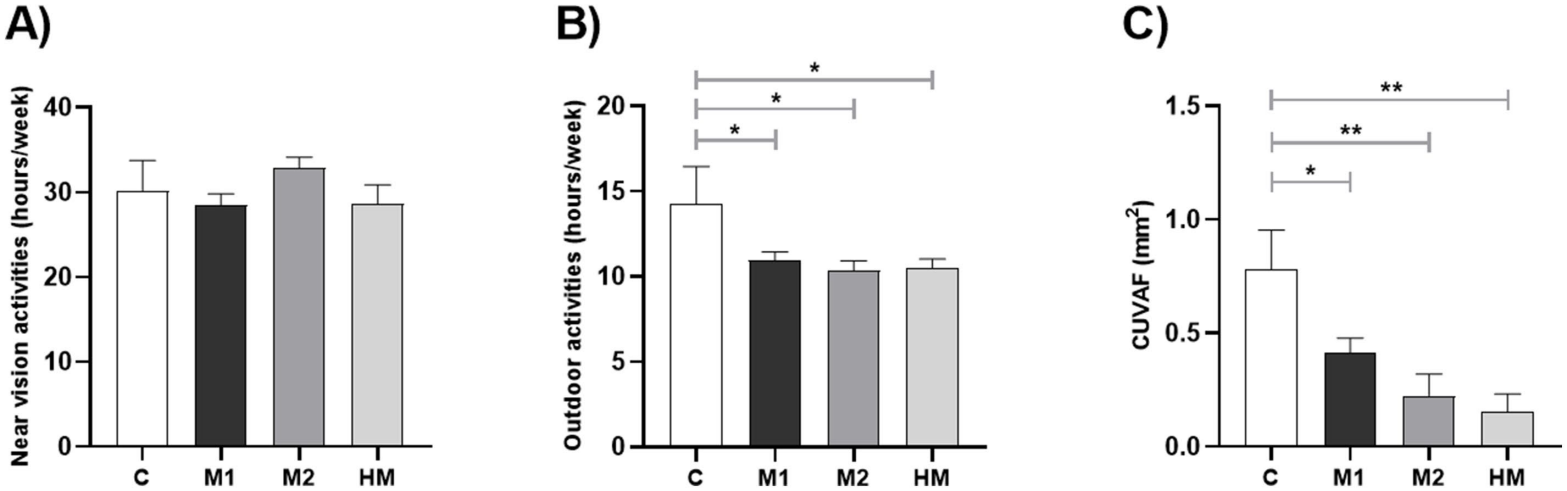
Analysis of the differences between the control and each myopic group (Controls vs. M1, M2 and HM). **(A)** Time spent doing near vision activities (hours/week). **(B)** Time spent doing outdoor activities (hours/week). **(C)** Differences in CUVAF-area (mm^2^). Values shown are the mean and standard error of the mean (SEM).*p < 0.05, **p < 0.01, ****p < 0.0001.

The data showed that there were 50 non-myopic subjects (19%) and 213 myopic subjects (81%), with 134 (51%) classified as M1, 51 (19%) as M2 and 28 (11%) as HM. The mean age of the subjects was 12.60 ± 2.82 years old, and 105 (69%) were female with no statistically significant differences between them in either case. However, the mean age of myopia onset was 8.23 ± 3.03 years old, which was significantly lower in M2 and HM group (6.86 ± 2.47 and 6.39 ± 2.62, respectively, *p* < 0.0001) compared to M1 (9.39 ± 2.67; Table 1; Figure 1B).

Finally, when differences in habits between male and female subjects regarding time spent doing NVA were analyzed, nonsignificant differences were obtained (*p* = 0.7287) being the mean 30.29 (±1.13) hours/week in the female group and 29.67 (±1.32) hours/week in the male group. On the contrary, data about OA shows that male subjects spent significantly more time outdoors than female subjects (*p* = 0.0429) being the mean 10.23 (±1.04) hours/week in the female group and 11.75 (±1.69) hours/week in the male group.

### Environmental factors

On average, the participants of the study spent 29.82 ± 10.97 h per week doing NVA and 10.81 ± 4.18 h per week doing OA. There were no significant differences in the time spent doing NVA between the myopic and control-group, but in relation to the OA, individuals from the M1, M2 and HM group spent significantly fewer hours per week outdoors than the control-group (*p* = 0.0252; Table 1; Figure 1).

### Conjunctival ultraviolet autofluorescence

The mean CUVAF-area of all the subjects in the study was 0.42 ± 0.85 mm^2^ (range 0–4.75 mm^2^). Dividing the sample into myopic and non-myopic, the mean of CUVAF-area was 0.33 ± 0.72 mm^2^ in the myopic-group and 0.78 ± 1.22 mm^2^ in the control-group. These differences were statistically significant between myopic and control group, showing that the mean CUVAF-area of all myopic-groups was significantly smaller in comparison to the control-group (*p* = 0.0023; Table 1; Figure 1).

Table 2 shows the distribution of CUVAF (mean and standard deviation) according to age groups with an age-CUVAF area correlation of r = 0.32 (*p* < 0.001; 95%CI 0.21–0.42) from which the formula for CUVAF area growth based on age, gender and geographic origin could be predicted:


$$\mathrm{CUVAF area}\;\left({\mathrm{mean total;mm}}^{2}\right)=K+\left(0.1^{*}Y\right)*{\mathrm{Age}}^{*}G$$

**Table 2:** Distribution of CUVAF (mean and standard deviation) according to age groups with an age-CUVAF area correlation of *r* = 0.32 (*p* < 0.001; 95% CI 0.21–0.42).

Age	*N*	CUVAF-area (mean ± SD)
5–11	100	0.12 ± 0.41
12–15	126	0.49 ± 0.92
>16	37	0.98 ± 1.16
Total	263	0.42 ± 0.85

SD, Standard Deviation.

**Figure 2:**
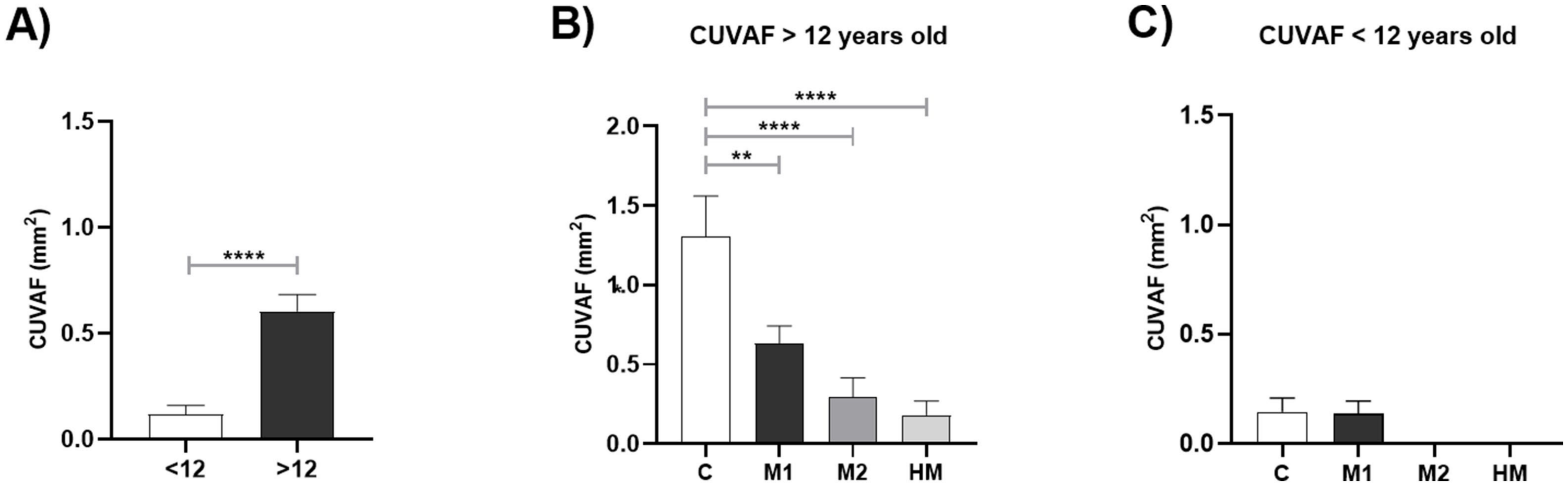
Graphical representation of differences in CUVAF-area (mm^2^) in groups according to age. **(A)** Under 12 years of age vs. over 12 years of age. **(B)** In individuals younger than 12 years, controls vs. M1, M2 and HM. **(C)** In individuals younger than 12 years, controls vs. M1, M2 and HM. Values shown are the mean and standard error of the mean (SEM). **p < 0.01, ****p < 0.0001.

Based on these results, participants were classified into two additional groups: ≥12 years old and < 12 years old. The mean CUVAF-area mean was 0.60 ± 1.00 mm^2^ in the older group and 0.12 ± 0.41 mm^2^ in the younger group, showing significant differences between both groups (*p* < 0.0001). Taking these differences into account, all the groups were separated by age, finding greater significant differences between groups in the participants older than 12 years old, in comparison to the global analysis (*p* < 0.0001; Figure 2).

**Figure 3:**
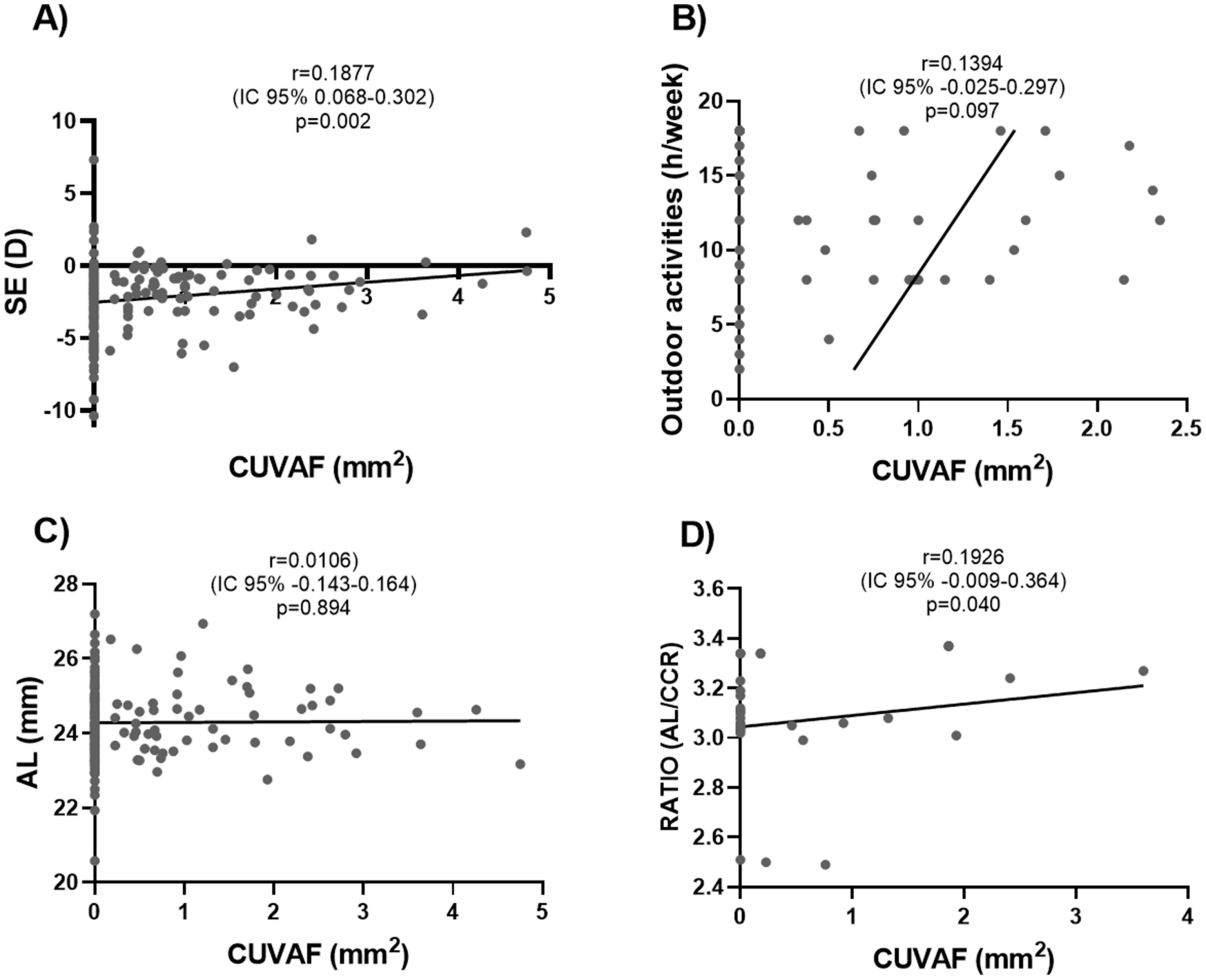
Pearson correlation analysis between CUVAF-area (mm^2^) and emmetropization-related ocular parameters. **(A)** Positive correlation between CUVAF and SE, with a statistically significant difference (*p* = 0.002). **(B)** No significant correlation was observed between CUVAF and AL (*p* = 0.894). **(C)** No significant correlation was proven between CUVAF-area and CCR (*p* = 0.372). **(D)** There is a positive correlation with statistically significant results between CUVAF and the obtained ratio from the AL and CCR (*p* = 0.040).

A Pearson correlation test was carried out, obtaining a significant inverse correlation between myopia degree and CUVAF-area (*p* < 0.002; *r* = 0.1877; CI 95%; 0.068–0.0302; Figure 3A). No clear correlation was found between CUVAF-area and AL or time spent doing OA, although a higher CUVAF-area trend was observed with the increase in time spent doing OA (*p* = 0.097; *r* = 0.1394; CI 95%; −0.025–0.297). (Figures 3B,C). In the case of AL, after adjusting the analysis for age, a clear inversely proportional trend is obtained, although without statistically significant results (*p* = 0.07; *r* = −0.14; 95% CI: −0.29 to 0.01). In order to a more complete analysis about AL and its interaction with the biometric measurements, we measured the correlation between CUVAF and the ratio obtained from the AL and CRC (*r* = 0.1394; CI 95%-0.025–0.297); *p* = 0.04 (Figure 3D.

**Figure 4:**
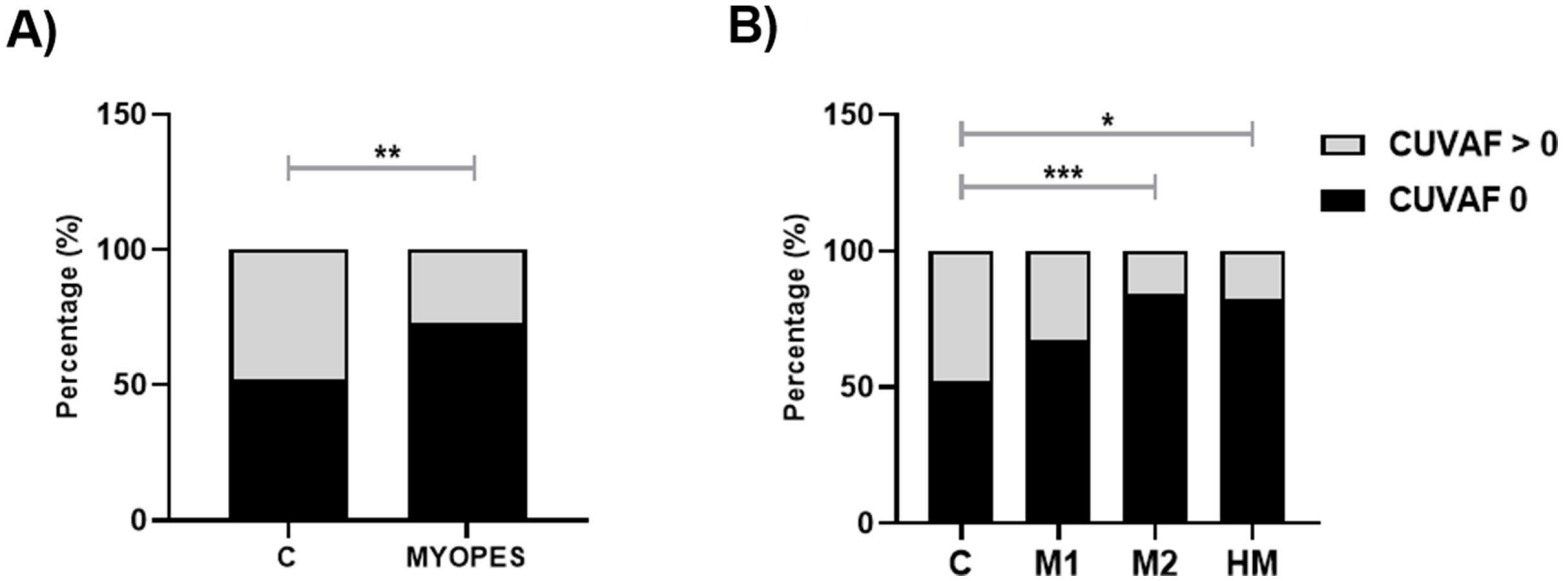
Percentage of participants with CUVAF-area (mm^2^) = 0. **(A)** Controls vs. myopes. **(B)** Controls vs. M1, M2 and HM. *p < 0,05; **p < 0,01.

The frequency of CUVAF-area absence (CUVAF-area = 0) was analyzed in each myopic and control-group, obtaining an odds ratio (OR) of 2.52 (CI 95%; 1.33–4.74), being considerably higher in the M2 and HM groups (4.96 (CI 95%; 1.96–12.58) and 4.25 (CI 95%; 1.40–11.35) respectively) compared to control-group. The differences in the percentage of patients with and without CUVAF were statistically significant between M2 and HM, and the control-group (*p* = 0.0006 and *p* = 0.0137, respectively; Figure 4). In the M1 group, an increasing trend in the percentage of no-CUVAF-area was found, without a statistically significant difference. (Figure 4B).

**Figure 5:**
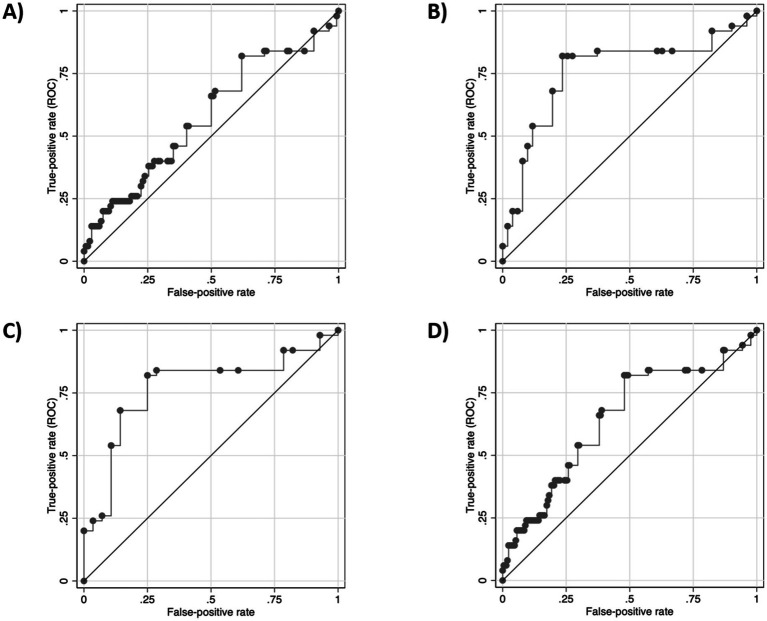
CUVAF discriminative capacity (total mean, mm^2^). **(A)** AUC of control vs. case group. **(B)** AUC of control vs. Low miopes (M1). **(C)** AUC of control vs. Moderate miopes (M2). **(D)** AUC of control vs. high miopes (HM).

Table 3 shows the discriminative ability of CUVAF (total mean) between myopic patients and controls. Between controls and myopic patients an AUC of 0.64 (95%CI 0.55–0.73) is obtained, with a sensitivity of 0.71 and a specificity of 0.52. When separating myopic patients into the different groups, a progressively higher AUC is obtained, reaching 0.77 (95%CI 0.66–0.88) in high myopic patients, also increasing its sensitivity to 0.89, with the specificity remaining unchanged (Figure 5; Table 3).

**Table 3:** Discriminative ability of CUVAF (total mean) between myopic patients and controls.

	AUC (IC 95%)	Sensitivity	Specificity
Myopes	0.64 (0.55–0.73)	0.71	0.52
M1	0.58 (0.48–0.67)	0.61	0.52
M2	0.75 (0.65–0.86)	0.88	0.52
HM	0.77 (0.66–0.88)	0.89	0.52

### Myopia family history

The questionnaire results showed that 42 (16%) of the children did not have any parent with myopia, 125 (48%) had at least one parent with myopia, and 69 (26%) had both parents with myopia (Table 1). The control-group presented the higher number of subjects without any myopic parent (29%, *p* < 0.01), while the percentage of subjects with both myopic parents was lower (20%, p < 0.01) compared with the myopic-groups (M1, M2 and HM; Table 1). However, the size of the CUVAF-area was analyzed according to family history obtaining a mean of 0.675 (±0.47) mm^2^ in the group of subjects with no parents with myopia, 0.468 (±0.66) mm^2^ in the group of those with only one parent with myopia, and 0.516 (±1.21) mm^2^ in the group of those who had two parents with myopia. Nevertheless, no significant differences were found between one or two parents with myopia and no parents with myopia group (*p* = 0.53 and *p* = 0.69 respectively; Supplementary Figure S1).

## Discussion

The growing importance of myopia due to the rapid increase in its prevalence (7, 47, 48) and its possible severe visual impairment (14, 15, 49), makes it necessary to further study its etiopathogenesis and the location of biomarkers that help us to control and monitor it, especially in the pediatric age group (15, 50). In this sense, our results show that CUVAF is a useful biomarker for exposure to OA, as well as its inverse relationship with the degree of myopia, confirming its usefulness also in the pediatric population in both aspects. In addition, a direct relationship between CUVAF and age is observed, being more frequent its appearance after 8–10 years of age, presenting a greater discriminative capacity in the second decade of life with an increase in its specificity and sensitivity. In this regard, a dynamic cut-off point could be established according to the age of the subjects by means of the formula explained above in the results, which implies a progressive increase of the CUVAF area throughout life, obtaining additional information that could help us to establish the risk of patients with myopia.

The rise in myopia prevalence cannot be attributed exclusively to genetic factors, indicating that environmental influences also play a crucial role in its development (51). Although NVA contribute to this trend (21–23, 52), the most significant modifiable risk factor is the reduction of OA, as one hour of OA reduces the risk of myopia by 13% (1). Most studies show the dose–response protective effect of OA on myopia onset (1, 5), and its influence on progression (6). In this sense, our study showed no significant differences in NVA between groups, as other age-group studies (6), but did find that the control-group engaged significantly more hours of OA compared to the myopic-group, particularly in the HM group, underscoring the protective effect of OA. It is well known that various ocular components undergo growth and maturation in younger children and the ocular growth patterns may be more sensitive to environmental influences during this period (1). In terms of NVA, the SAVES study describe that they may have an impact on the early development of myopia, but only in young children (10, 53).

Regularly, the relationship between OA and the degree of myopia has been measured by lifestyle questionnaires which are prone to recall bias, making them an inaccurate method (41). This situation is exacerbated in the pediatric population, since these questionnaires must be filled out by a third person (legal-guardians) due to the age of the patients. Consequently, it is important to evaluate CUVAF as a biomarker for OA at the early age of myopia onset, as most studies have focused on older populations. In our study both questionnaire data on OA hours and CUVAF-area show statistically significant differences between controls and myopia groups. However, the measurement of CUVAF-area showed bigger statistical significance and more sensitivity. In this way, the results showed that having CUVAF-area protect to myopia 2.5 times, being almost 5 times the protection in case of HM. Additionally, when correlating these two measures, OA hours an CUVAF-area, although no statistically significant correlation was found, a clear trend was observed supporting the theory that the biomarker is more objective and more powerful predicting a lower risk of myopia development.

Since the direct relationship of CUVAF with OA is also observed in children, it is reasonable to consider its usefulness as a protective marker for myopia in this age group (34, 39, 48). Our results show significant differences between both groups, with the CUVAF-area being significantly higher in the control-group than in the group of myopic patients. A statistically significant difference is obtained when we separate the groups according to the degree of myopia, demonstrating how the CUVAF-area decreases as the degree of myopia increases. In the clinical practice these changes in the relative risk could play an interesting role, the simply describing the presence or absence of CUVAF could help us identify the children at higher risk that could benefit from a closer follow up and early treatment.

An interesting observation is that this relationship increases in the subgroup of children older than 12 years, which could imply that CUVAF may have a certain dynamic or cumulative component, or that this increase may be due to the difference in behavior of children of different ages, being more exposed to OA from the age of 12 years.

In our environment, we are constantly exposed to UV radiation, and its relationship to changes in the ocular surface is well known (54), as well as the alterations of proteins which provide these cells the ability to autofluorescence when excited by UV radiation (41, 54). Some researchers have observed that the CUVAF-area increased in intensity and surface-area, especially in subjects who do a lot of OA, confirming the theory of the cumulative effect of UV radiation (55). These data, which have been verified in different regions of the world (33), show the usefulness of CUVAF for the follow-up of ocular pathologies and adherence to lifestyle changes in patients (54–58).

Axial length is a primary factor linked to myopia development, and therefore, numerous studies examining CUVAF in both adults and children have sought to correlate CUVAF-area with axial length. However, to our knowledge, including the results of this study, none have found a significant correlation, despite a notable correlation with spherical equivalent. The absence of this correlation with axial length may be due to its dependence on several factors such as height, sex and age of the subjects. In our study, an adjustment for age and a ratio (AL/CCR) has been performed, with the intention of eliminating these confounding factors, thus obtaining a partial CUVAF-AL correlation (adjusted for age) and significant in the case of the ratio AL/CCR.

This has led to the hypothesis that light exposure influences not only axial length but also the balance of optical power during emmetropization. Consequently, CUVAF has correlated not only with axial length but also with corneal curvature radius and an index reflecting the relationship between these parameters (AL/CCR). Interestingly, this correlation between CUVAF and AL/CCR is statistically significant; suggesting that sunlight exposure indeed affects the balance between ocular optical power and axial length, encompassing both refractive and axial myopia origins. It could be interesting to evaluate this parameter in a larger sample, and even in adult population, since its possible impact has not been previously analyzed according to the current bibliography.

As the results of this study have shown, the implication of genetics in the development of myopia is clear, especially in those patients with an early onset of the disease. However, the results also manifest that in older pediatric patients (over 12 years of age) the environmental component plays a very important role that must be taken into account.

It could be hypothesized that parents with myopia have a lifestyle that favors this increase in myopia (more NVA and few OA), and that this is transmitted to their children, thus favoring an increase in myopia with a certain environmental, rather than genetic, character. Nevertheless, the data obtained have shown no significant differences in the size of the CUVAF-area according to family history (Supplementary Figure S1), which leads to the conclusion that the habits and lifestyle of the patients are not necessarily due to the existence of a family history of myopia.

The main limitation of this study is that most of the subjects were obtained from the Department of Ophthalmology, hence there are fewer controls and more myopic children. On the other hand, the strengths of this study are its large sample size, as well as the homogeneity of the sample in terms of geographical and socioeconomic status, so NVA and OA were the only differential variables regarding environmental risk factors. Even so, it would be necessary to do more studies with a larger sample size and in different environments.

## Conclusion

The results of this study prove that CUVAF is a useful biomarker for exposure to OA, as well as its inverse relationship with the degree of myopia, confirming its usefulness also in the pediatric population in both aspects, especially in the second decade of life. In addition, a direct relationship between CUVAF and age is observed, being more frequent its appearance after 8–10 years of age, presenting a greater discriminative capacity in the second decade of life with an increase in its specificity and sensitivity. Future works are needed to consolidate the capacity of this biomarker to differentiate which patients may have a higher risk of developing myopia during the growing age, being able to provide individualized treatments based on that risk, as well as to assess adherence to the behavioral measures that may be proposed in the clinical practice as a first line of action for the treatment of childhood myopia.

## Data Availability

The raw data supporting the conclusions of this article will be made available by the authors, without undue reservation.
