# Supplementary figures and images for: Conjunctival ultraviolet autofluorescence as a biomarker of outdoor time in myopic children
Source: Front Med (Lausanne). 2024 Dec 9;11:1492180. doi: 10.3389/fmed.2024.1492180 (PMC11663684; doi:10.3389/fmed.2024.1492180)

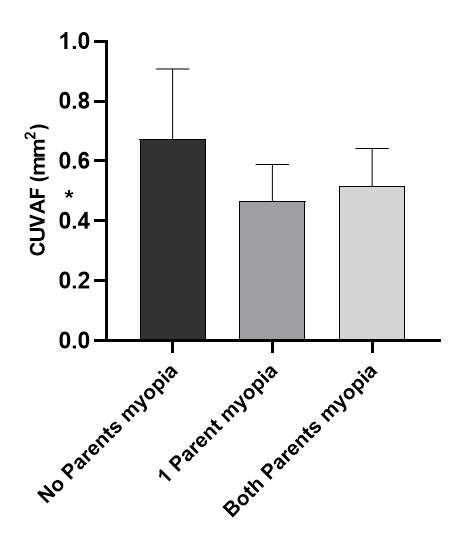

Supplement: Supplementary file 1 [file Image_1.TIF]
